# Berberine-loaded iron oxide nanoparticles alleviate cuprizone-induced astrocytic reactivity in a rat model of multiple sclerosis

**DOI:** 10.1007/s10534-024-00648-4

**Published:** 2024-11-14

**Authors:** Ghadha Ibrahim Fouad, Mostafa Mabrouk, Sara A. M. El-Sayed, Mohamed F. Abdelhameed, Maha Z. Rizk, Hanan H. Beherei

**Affiliations:** 1 Department of Therapeutic Chemistry, Pharmaceutical and Drug Industries Research Institute, National Research Centre, 33 El-Bohouth St., Dokki, Cairo, 12622 Egypt; 2 Refractories, Ceramics and Building Materials Department, Advanced Materials, Technology and Mineral Resources Research Institute, National Research Centre, 33 El Bohouth St., Dokki, PO Box 12622, Cairo, Egypt; 3 Pharmacology Department, Medical Research and Clinical Studies Institute, National Research Centre, 33 El-Bohouth St., Dokki, Cairo, 12622 Egypt

**Keywords:** Berberine, Cuprizone, Iron oxide nanoparticles, Neurotoxicity, S100β, GFAP

## Abstract

Berberine (BBN) is a naturally occurring alkaloid as a secondary metabolite in many plants and exhibits several benefits including neuroprotective activities. However, data on the neuromodulating potential of nanoformulated BBN are still lacking. In the present study, BBN loaded within iron oxide nanoparticles (BBN-IONP) were prepared and characterized by transmission electron microscopy FTIR, X-ray photoelectron spectroscopy particle-size distribution, zeta potential, and HPLC. The remyelinating neuroprotective potential of BBN-IONP relative to free BBN was evaluated against cuprizone (CPZ)-induced neurotoxicity (rats administered 0.2% CPZ powder (w/w) for five weeks). CPZ rats were treated with either free BBN or IONP-BBN (50 mg/kg/day, orally) for 14 days. Cognitive function was estimated using Y-maze. Biochemically, total antioxidant capacity lipid peroxides and reduced glutathione in the brain tissue, as well as, serum interferon-gamma levels were estimated. Moreover, the genetic expression contents of myelin basic protein Matrix metallopeptidase-9 Tumor necrosis factor-α (TNF-α), and S100β were measured. The histopathological patterns and immunohistochemical assessment of Glial Fibrillary Acidic Protein in both cerebral cortex and hippocampus CA1 regions were investigated. CPZ-rats treated with either free BBN or IONP-BBN demonstrated memory restoring, anti-oxidative, anti-inflammatory, anti-astrocytic, and remyelinating activities. Comparing free BBN with IONP-BBN revealed that the latter altered the neuromodulating activities of BBN, showing superior neuroprotective activities of IONP-BBN relative to BBN. In conclusion, both forms of BBN possess neuroprotective potential. However, the use of IONPs for brain delivery and the safety of these nano-based forms need further investigation.

## Introduction

Multiple sclerosis (MS) is an inflammatory neurodegenerative and demyelinating disorder of the central nervous system (CNS) (Lassmann et al. 2014), therapeutic options to prevent demyelination are limited (Larochelle et al. 2016). The underlying neuro-pathoetiology of MS is obscure; however, oxidative stress, microglial and astrological activation, and neuroinflammation are well-defined mechanisms for axonal loss and demyelination in MS (Largani et al. 2019; Zirngibl et al. 2022). Cuprizone (CPZ), a copper chelator, provokes a well-defined and highly reproducible pattern of non-autoimmune-mediated demyelination (Praet et al. 2014). CPZ provokes the mitochondrial dysfunction of myelin-forming cells, impairs oligodendrocyte function, induces demyelination in several brain regions, and has been found to nearly resemble “pattern-III lesions” in MS-patients, from the histopathological aspect (Clarner et al. 2012; Nicola et al. 2024).

CPZ, after 15 days of administration, can consistently cause demyelination and damage to the myelin sheath, making it a traditional model of MS (Adamo et al. 2006). CPZ-associated neurotoxicity affects both genders of rodents, but the effect is more aberrant in males, CPZ-induced rodent model of demyelination could represent an excellent MS experimental model to study CPZ-induced neurotoxicity and peripheral neuropathy (Ünsal and Özcan 2018), and to investigate myelin protection and remyelination after drug intervention (Yamamoto et al. 2014). The blood–brain barrier (BBB) protects the brain, preventing several therapeutic molecules from reaching a pharmacologically effective level in the brain, rendering the development of neurotherapeutic agents for MS a great challenge (Ballabh et al. 2004).

Berberine (BBN), a natural isoquinoline alkaloid, exhibited several therapeutic activities including neuroprotection; through exerting anti-inflammatory and anti-oxidative functions (Ahmed et al. 2015; Wang et al. 2019; Ibrahim Fouad and Ahmed 2021a, b). BBN exhibited neuroprotective potential against several experimentally induced neurotoxicity in animal models (Soudi et al. 2019; Ge et al. 2023; Gendy et al. 2023; Tian and Sharma 2023; Tseng et al. 2023; Wang et al. 2023a; Mehrjerdi et al. 2024). However, its poor oral bioavailability and limited potential to cross the BBB hinder its neurotherapeutic efficiency; therefore several studies aimed at improving the neurotherapeutic activities of BBN through developing novel molecular derivatives of BBN (Mirhadi et al. 2018; Koly et al. 2023), BBN-loaded nanoparticles (NPs) (Leimann et al. 2023), or liposome encapsulation of BBN (Wang b et al. 2023). This could be ascribed to the fact that BBN is more appropriate for long-term therapeutic application when it’s delivered in the form of liposome, microsphere, and nanostructures to improve the brain uptake of BBN; therefore the preparation of suitable and safe BBN dosage forms is crucial for the treatment of neurological disorders (Sunhe et al. 2024). For instance, the neuroprotective potential of different types of BBN-loaded NPs were investigated such as BBN-bovine serum albumin NPs (Attia et al. 2024); BBN-loaded solid dispersions (SD) that could act as effective acetylcholinesterase inhibitors (Leimann et al. 2023), BBN-loaded zein/hyaluronic acid composite NPs, and Poly Lactic-*co*-Glycolic Acid (PLGA)-encapsulated BBN NPs that effectively demonstrated anti-epileptic potential in animal models (El-Nahas et al. 2023; Saha et al. 2023; 2024).

The most utilized inorganic NPs in biomedical applications, especially for drug delivery, are iron oxide nanoparticles (IONPs), the magnetite (Fe_3_O_4_) form. It is widely used in the biological applications and has diameters ranging from 1 to 100 nm (Ibrahim Fouad et al. 2023; El-Sayed et al. 2024). There is an interest in the synthesis of NPs with magnetic properties, such as IONPs, due to their distinct physicochemical characteristics (Zhu and Diao 2011). Magnetite Fe_3_O_4_ is the most widely used magnetic material in many fields, despite the existence of other varieties such as iron oxide, metal ferrite, alloys, nickel, and cobalt, because of its chemical stability, super paramagnetic property, high biocompatibility, non-toxicity, inertness, and ease of detection in the human body (Ibrahim Fouad et al. 2023). Because of its small size, biocompatibility and large surface area, magnetite Fe_3_O_4_ is the most frequently utilized magnetic nanomaterial as a nanocarrier (Prabhakar et al. 2011). Because they provide the least risk among metallic NPs, IONPs are used in biomedicine; this might be attributed to their limited biodistribution of IONPs as they are subjected to body’s highly active clearance mechanism (Albanese et al. 2012; Valdiglesias et al. 2016). IONPs have a variety of biomedical uses, such as biosensing, enzyme immobilization, tissue engineering, cell labeling, enzyme immobilization, protein purification, magnetic hyperthermia and contrasting agents (CA) for magnetic resonance imaging (MRI) (Ibrahim Fouad et al. 2023). Additionally, IONPs can cross BBB and target a specific area of the body when exposed to an external magnetic field (Ibrahim Fouad et al. 2023).

Toxicological investigation of novel nanomaterials is an essential step towards their clinical approval (Ezealigo et al. 2021); magnetite NPs are generally nontoxic and proven to prevent protein aggregation (Bellova et al. 2010). Iron oxide is the most studied biomaterial as FDA-approved nano-based therapeutics (Bobo et al. 2016). The particle size, morphology, and magnetic features of IONPs controls their biomedical applications (Ezealigo et al. 2021); magnetic IONPs have found to be effective as contrast agents, drug carriers, and thermal-based nanotherapeutics (Arias et al. 2018; Montiel Schneider et al. 2022). However, they are restricted for some clinical applications by low solubility and toxicity (Bobo et al. 2016; Manshian et al. 2017; Arias et al. 2018); therefore extensive in vitro and in vivo trials regarding their toxicity and biocompatibility are required before clinical approval.

In this study, we employed IONPs to load and deliver BBN to a rat model of CPZ-induced neurotoxicity and demyelination; IONPs present promising potentials in the development of nanodrugs; due to their unique physicochemical features and magnetic properties (Elbialy et al. 2019). Recent progress in synthesis, characterization, and surface modification of the IONPs has improved their efficacy as drug carriers (El-Boubbou 2018; Ibrahim Fouad and Rizk 2024); such as using IONPs to enhance the bioavailability of bioactive molecules such as polyphenols (Aboushoushah et al. 2021; Ibrahim Fouad et al. 2022; 2023).

This study aimed to evaluate the potential of BBN-IONP as a nano-neurotherapeutic agent against CPZ-induced neurotoxicity, and to compare its neuroprotective activity to that of free BBN; through investigating different biochemical markers (TAC, LPO, GSH, and IFN-γ), in addition, we evaluated the expression of a selected panel of mRNAs as molecular biomarkers (MBP, MMP-9, TNF-α, and S100β) that are implicated in demyelination, neuroinflammation, and astrocytosis in CPZ-induced rats. Moreover, the goal of our present study is to identify the roles of S100β and GFAP in mediating the neurotoxic astrocytic activity in CPZ-rats.

## Materials and methods

### Materials

The chemicals used for preparing iron oxide (Fe_3_O_4_) nanoparticles (IONPs) included anhydrous iron chloride (FeCl_3_) with a purity of 97%, a molecular weight of 162.20 g/mol, and sourced from Sigma-Aldrich in Germany. Ethylene glycol (CH_2_OH) _2_ with a purity of 99%, a molecular weight of 62.07 g/mol, and sourced from Alpha in India. Hydrated hydrazine (N_2_H_4_.H_2_O) with a purity of 99%, a molecular weight of 50.06 g/mol, and sourced from Advent in India. Lastly, Absolute ethanol (C_2_H_5_O) with a purity of 99.8%, a molecular weight of 46.07 g/mol, and sourced from Piochem, Egypt.

### Method of preparation

**Fig. 1:**
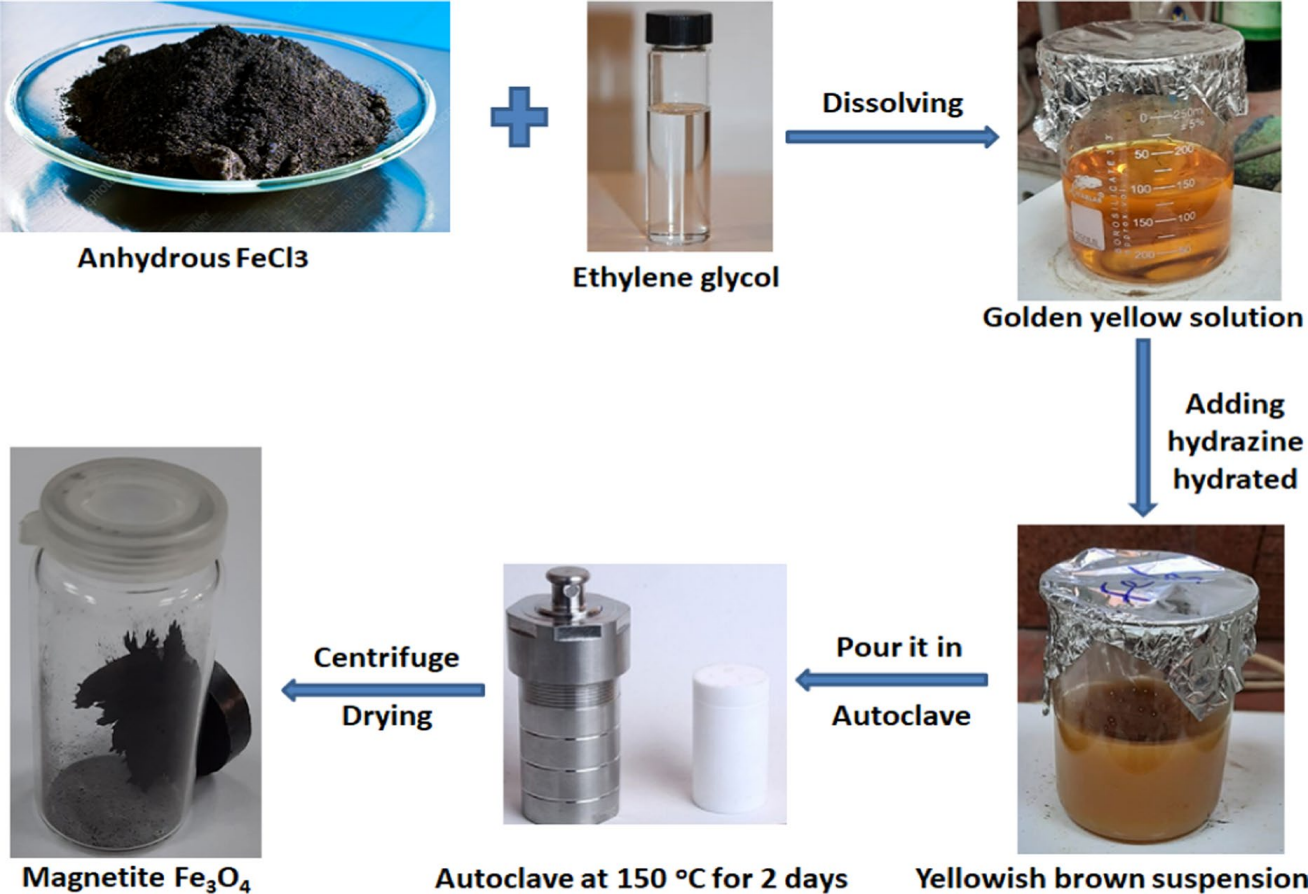
Preparation method of iron oxide (Fe_3_O_4_) nanoparticles (IONPs)

With some modifications Fe_3_O_4_ nanoparticles (IONPs) were prepared according to Huang et al. (2011); where anhydrous iron chloride (0.32 g) was added to ethylene glycol (60 ml) with continues stirring until FeCl_3_ completely dissolved and the golden yellow color appeared. Subsequently, 6 ml of hydrated hydrazine was introduced into the preceding solution while stirring persistently till the solution transformed into a yellowish-brown suspension. The prepared suspension was transferred into an 80 ml Teflon-lined stainless-steel autoclave and subjected to drying in a dryer at a temperature of 150 °C for duration of 48 h. By using the centrifuge at certain conditions (7000 rpm, 15 min, and 20 °C); the precipitate was collected by washing it three times with water and one time with ethanol. Finally, IONPs were obtained by drying at 37 °C for 2 days (Fig. 1.).

### Characterization techniques

#### FTIR analysis

To determine the functional groups present in the synthesized IONPs and BBN-loaded Fe_3_O_4_-NPs (IONP-BBN), we acquired infrared spectra using a Fourier Transform Infrared (FTIR) spectrophotometer (model FT/IR-6100 type A). The spectra were obtained within a wave number range spanning from 400 to 4000 cm^−1^. The collected samples were produced by combining them with KBr.

#### TEM analysis

The particle size of IONPs was determined by Transmission Electron Microscopy (TEM) (JEOL, Japan, JEM-2100, ELECTRON MICROSCPE, TEM-HR) before and after BBN loading. IONPs free and BBN-loaded suspensions were produced using ethanol. Subsequently, 5 μl of each suspension was applied onto a copper grid and TEM images were captured after the grids were dried in air.

#### Particle size distribution and zeta potential

The size distribution and zeta potential of IONPs, both before and after BBN loading, were measured by light scattering with a Zetasizer Nano ZS instrument (Malvern Instruments, UK) equipped with a 633 nm laser. The samples were evenly distributed in a transparent disposable zeta cell by utilizing deionized water at 25 °C. The site for measuring the size distribution is located 5.50 mm away from the wall of the Zeta cuvette, while the place for measuring the zeta potential is 2.00 mm away. The data analysis and measurement of zeta potential were performed using Malvern Instrument’s dispersion technology program, namely Version 7.13.

#### X-Ray photoelectron spectroscopy (XPS)

XPS was used to confirm the loading of the BBN on IONPs through the determination of the functional groups. The measurements were collected using a SPECS spectrometer apparatus (XR-50 source with monochromator-FOCUS 600) and Al Kα monochromatic radiation with an energy of 1486.7 eV. A hemispherical analyzer was employed for this purpose. A pressure of 1109 mbar was applied for the ultrahigh vacuum (UHV). The sample was affixed to a sample holder using a carbon tape that had a double-sided adhesive. The data was statistically analyzed using Casa XPS processing and ULVAC-PHI MultiPak software (ver. 9.0.1, Osaka, Japan).

#### Drug loading and HPLC analysis

The drug loading of the BBN active molecules was loaded on magnetite IONPs, by direct dissolving of BBN in phosphate buffer saline (PBS) and subsequently IONPs were suspended in drug solution using ultrasonic bath with ratio (1:1). The concentration of BBN in the final formulation was estimated to be (12 mg/ml). HPLC analysis was used to evaluate the BBN concentration through in vitro release. The in vitro release of BBN from BBN-loaded Fe_3_O_4_-NPs was carried out in PBS at 37 °C. Briefly, the BBN-loaded IONPs were put in dialysis bag, which was dipped in plastic containers filled with 50 ml of PBS. At certain time intervals (2, 4, 6, 24, 72, 168, 336 and 672 h), 3 ml of PBS was withdrawn from the container and replaced by another 3 ml of fresh PBS. The concentrations of BBN were determined by injecting 20 μl of each sample onto an Agilent Zorbax Eclipse Plus C18 column (4.6 mm × 250 mm i.d., 5 μm). The experiment was conducted under the specified conditions: The mobile phase consisted of a mixture of ACN (acetonitrile) and 20 mM phosphoric acid at a ratio of 40:60 (^v^/_v_). The flow rate of the mobile phase was set at 1.2 mL/min. The column temperature was kept constant at 35 °C. The MWD (maximum wavelength detection) was set to 346 nm. An Agilent 1260 series was used for HPLC analysis. Each sample was measured three times to determine the concentrations.

#### In vivo study

##### Chemicals

Berberine chloride hydrate (technical, ≥ 90% AT) was bought from Sigma Aldrich (St. Louis, MO, USA) and dissolved in phosphate buffer saline (PBS, 0.1 M; pH 7.4) purchased from Sigma Aldrich (St. Louis, MO, USA). Cuprizone (CPZ) was bought from Sigma, St. Louis, MO, USA. Interferon-γ (IFN-γ) levels were estimated using commercially available ELISA kits according to manufacturer’s instruction (Quantikine, Minneapolis, USA). GFAP immunoexpression was estimated using commercially available kits according to manufacturer’s instruction (Cat. No. 13–0300; Thermo scientific Co. 1:100). Malondialdehyde (MDA), glutathione reduced (GSH), and total antioxidant capacity (TAC) were determined colorimetrically using Biodiagnostic assay kits, Egypt. QuantiTect real time-PCR Kits and RNeasy Kits were purchased from QIAGEN, Germany. Primers of MBP, S100β, TNF-α, and MMP-9 were purchased from Qiagen (Germany).

##### Animals

Forty adult male Wistar rats weighing 120–140 g (8-weeks) were provided by the Animal House of the National Research Center (NRC), Egypt. The rats were maintained with constant 12 h/12 h dark and light cycle at room temperature (22 ± 3 °C) throughout the experimental period. The animal protocol was adopted in accordance with the National Research Council’s Guide for the Care and Use of Laboratory Animals (NIH Publications No. 8023, revised 1978), and experimental procedures were approved by the Ethical Committee, National Research Centre (NRC), Egypt (Approval No. 19–313).

##### Demyelination induction and treatment

To induce neurotoxicity and demyelination, rats were fed with milled forage mixed with 0.2% CPZ powder (w/w) for 5 consecutive weeks followed by one further week of normal chow feeding. The 5-week induction system was followed according to Omotoso et al. (2018).

Oral administration of BBN or IONP-BBN (50 mg/kg body weight) started on the 1st day of the 6th week and lasted for 14 days. CPZ and BBN or IONP-BBN treatment overlapped for one week to make sure that BBN and IONP-BBN were received and metabolized while CPZ was still present.

##### Experimental design

The rats were divided into 4 groups (n = 10) as follows:

Group 1 Negative control group: rats were fed a normal diet for the entire period.

Group 2 CPZ-neurotoxicated group: rats received 2% CPZ milled diet only for 5 weeks.

Group 3 CPZ + BBN group: rats received a combination of 0.2% CPZ-diet and an oral dose of free BBN (50 mg/kg) daily for 14 days (Ibrahim Fouad and Ahmed 2021b).

Group 4 CPZ + IONP-BBN treated group: rats received a combination of 0.2% CPZ milled diet and an oral dose of IONPs-BBN (50 mg/kg) daily for 14 days. IONPs dose (100 mg/kg) was estimated in our previous study (Mabrouk et al. 2021).

##### Behavioral test: Y-maze test for assessment of spatial working memory

Y-maze can be used to evaluate short-term memory, according to Kraeuter et al. (2019). Spatial working memory or spontaneous alternation involves interaction across the hippocampus and the prefrontal cortex (Sarnyai et al. 2000). The consecutive entries into all three arms of the Y-maze are recognized as “alteration”. The number of alternations and arm entries was registered to calculate the “% of alternation”; a high % of alternation signifies a good working memory. The % of alternation was calculated according to this formula:

“% of Alternation= (Number of Alternations/ [Total number of arm entries−2]) ×100”

##### Spatial reference memory

The test involves placing the rat into the Y-maze and blocking one arm of the maze during the "training session." After a specific time interval, known as the inter-trial interval (ITI), the rat was located back with the blockage removed “Testing session”. The length of the ITI is directly proportional to the memory load. The degree of spatial memory could be estimated by counting the number of entries into the novel arm and then comparing it to the entries into the other arms. Impaired spatial memory indicates dysfunction of the hippocampus and is recognized during the “Testing session”, by the rat demonstrating a lack of preference for any of the arms.

##### Brain tissue collection and processing

At the end of the experimental period, the deeply anaesthetized rats were subjected to cervical dislocation. The brain were rapidly excised, washed with saline (0.9% sodium chloride), and divided longitudinally into two halves. Brain was dissected out; one portion of the brain was homogenized in four-volume of cold PBS (0.1 M; pH 7.4). After centrifuging at 3000 rpm for 15 min at 4 °C, the clear supernatant was pipetted. The other portion of the brain was used for histopathological and immunohistochemical analyses. Samples of fresh brains were preserved in liquid nitrogen at − 80 °C for molecular assays.

##### Biochemical analyses

Biochemical assays in serum and brain homogenate were conducted on a UV/Vis Spectrophotometer (Aglient Technologies, India).

##### Estimation of serum total antioxidant capacity (TAC) levels

Serum TAC levels were estimated according to Koracevic et al. (2001), by an enzymatic reaction that results in a colored end-product that could be estimated colorimetrically at 505 nm.

##### Brain Lipid peroxidation (LPO) content

Lipid peroxidation in the brain was assessed according to Ohkawa et al. (1979), through estimating malondialdehyde (MDA) spectrophotometrically at 534 nm. The data were expressed as nmol/g tissue.

##### Brain reduced glutathione (GSH) content

Brain GSH content was estimated according to Beutler et al. (1963), through spectrophotometric detection of the yellow colored end-product; due to the reaction between GSH and DTNB “5, 5′-dithiobis-(2-nitrobenzoic acid)”, at 405 nm. The results were expressed as mg/g tissue.

##### Determination of interferon-gamma (IFN-γ) levels

Serum IFN-γ levels were assayed by ELISA kit as described in the manufacturer’s guidelines. The results expressed as *p*g/ml protein.

##### Quantitative real time qPCR (qRT-PCR) analysis

Genetic expression contents of myelin basic protein (MBP), Matrix metallopeptidase-9 (MMP-9), Tumor-necrosis factor α (TNF-α), and S100β calcium-binding protein (S100β) were estimated by quantitative reverse transcriptase polymerase chain reaction (qRT-PCR) in the brain samples. Total RNA was extracted using the standard TRIzol® Reagent extraction method (cat#15,596–026, Invitrogen, Germany), according to the manufacturer’s guidelines. Complementary DNA (cDNA) and mRNA expression of MBP, MMP-9, TNF-α, and S100β, were conducted using one-step QuantiTecht SYBR green qRT-PCR Master Mix (Qiagen; USA; Cat No. 204243). The housekeeping gene, β-actin, was used as an internal reference. The sequences of primers are described in Table 1. The relative quantification was estimated by using the 2^*−*ΔΔCT^ method.

**Table 1:** Sequence of the used primers

Gene name	Primer sequence (5’ → 3’)
MBP	Forward 5’- CCATCCAAGAAGACCCCACA-3’ Reverse 5’- CCCCTGTCACCGCTAAAGAA-3’
S100β	Forward 5’—GAGAGAGGGTGACAAGCACAA-3’ Reverse 5’-GGCCATAAACTCCTGGAAGTC-3’
TNF-α	Forward 5’- TACTGAACTTCGGGGTGATTGGTCC-3’ Reverse 5’-CAGCCTTGTCCCTTGAAGAGAACC- 3’
MMP-9	Forward 5’- AGGTGCCTCGGATGGTTATCG-3’ Reverse 5 -TGCTTGCCCAGGAAGACGAA-3’
β-Actin	Forward 5’-ACCCACACTGTGCCCATCTA- 3’ Reverse 5’- CGGAACCGCTCATTGCC-3’

*MBP* myelin basic protein, *MMP-9* Matrix metallopeptidase-9, *TNF-α* Tumor-necrosis factor α, and *S100β* S100β calcium-binding protein

##### Histopathological investigations

Sections of the brain (cerebral cortex and hippocampus CA1 region) were fixed in 10% neutral buffered formalin for 72 h. Samples were processed in serial grades of ethanol, cleared in Xylene; infiltrated, and embedded into Paraplast media. Sections of 4 μm were cut by rotatory microtome. Brain tissue sections were stained with hematoxylin and eosin (H&E) then examined by using a light microscope (Leica Microsystems GmbH, Germany). Those standard procedures were performed according to Culling (2013).

##### Immunohistochemistry: glial fibrillary acidic protein (GFAP)

Deparaffinized retrieved brain tissue sections of 5 μm-thickness were treated with 0.3% H_2_O_2_ for 20 min. Brain samples were incubated with anti-glial fibrillary acidic protein by using a monoclonal antibody (1:100), overnight at 4 °C. Sections were washed out by PBS and incubated with the secondary antibody HRP Envision kit (DAKO) for 20 min; then washed out and incubated with diaminobenzidine (DAB) for 15 min. Sections were washed with PBS then counterstained with hematoxylin, dehydrated, cleared in xylene, and coverslipped for microscopic examination. According to Abbas et al. (2021); six randomly selected non-overlapping fields were selected and scanned from the cerebral cortical regions and hippocampal CA1 regions of each sample for the determination of immunostaining area % of GFAP using image analysis software (Image J, version 1.46a, NIH, Bethesda, MD, USA).

##### Statistical analysis

The collected data was coded and tabulated using Statistical Package for Social Science (SPSS 24 for windows; SPSS Inc, Chicago). The analysis was performed using a one-way analysis of variance (ANOVA), followed by Duncan’s post-hoc test. The data were expressed as mean ± SEM (standard error of the mean). Statistical significance was defined at the *p*-value < 0.05.

## Results

### Characterization of the synthesized IONPs

#### FTIR analysis

**Fig. 2:**
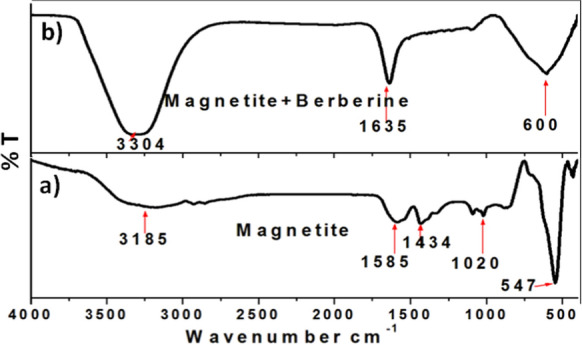
FTIR spectra of **a** IONPs and **b** BBN-loaded IONPs (IONP-BBN)

The Fourier Transform Infrared (FTIR) spectrum of the synthesized IONPs is depicted in Fig. 2a. The prominent band at 547 cm^−1^ corresponds to the vibration modes of the Fe–O bond, which is the distinctive absorption peak of magnetite Fe_3_O_4_. The band observed at 1020 cm^−1^ is associated with the stretching of C–O bonds in alcoholic derivatives. The distinct peak at 1434 cm^−1^ in every spectrum corresponds to the bending of C-H bonds. The prominent peak observed at 1585 cm^−1^ in all spectra is attributed to the O–H group of the ethylene glycol precursor. The prominent peak observed at 3185 cm^−1^ is ascribed to the stretching of O–H bonds, which can be attributed to the presence of an alcoholic group (ethylene glycol) (Mabrouk et al. 2021). Figure 2b showed a beak at 3304 cm^−1^ might be corresponding to C-H stretching vibrations of methoxy group in BBN (Sreeja and Nair 2018).

#### TEM analysis of berberine-loaded Fe_3_O_4_-NPs (IONP-BBN)

**Fig. 3:**
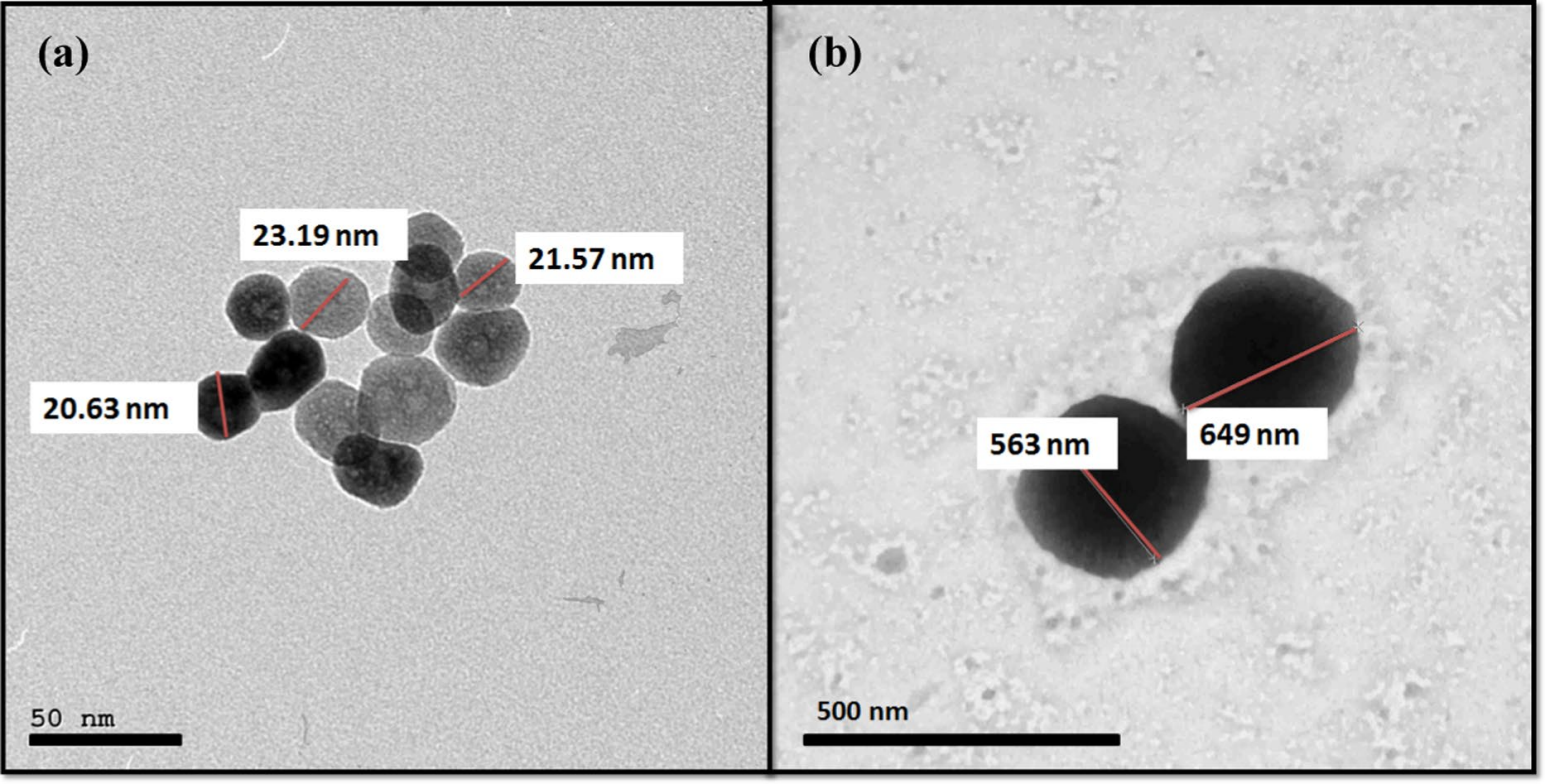
TEM Images of **a** Magnetic iron oxide nanoparticles (IONPs) and **b** BBN-loaded iron oxide nanoparticles (IONP-BBN)

TEM examination was used to assess the synthesized IONPs’ morphology and architecture both before and after BBN loading (Fig. 3a). The morphology of the synthesized IONPs were revealed by TEM imaging to have a form resembling nanosphere particles with a diameter ranging from 21 to 23 nm. Figure 3b showed the IONP-BBN and showed how their size changed to (563–649) nm. The IONP-BBN shows varying degrees of illumination, which suggests the creation of a core–shell formulation type. The darker zone (the center portion) is ascribed to the IONPs and the lighter area (the outer limits) to BBN.

#### Particle size distribution and zeta potential of (IONP-BBN)

**Fig. 4:**
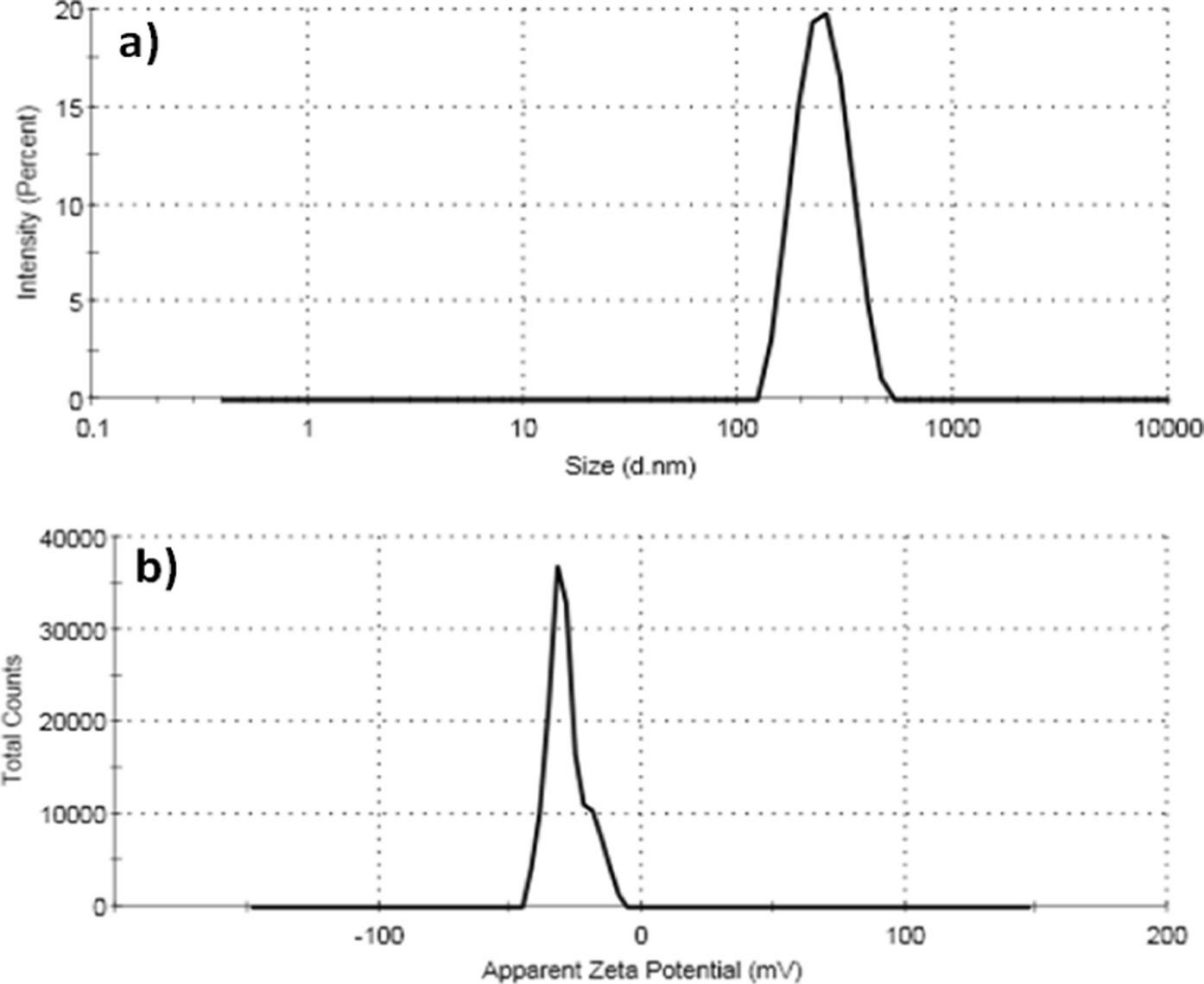
**a** Particle size distribution curve of the prepared IONP-BBN, and **b** Zeta potential profile of the prepared IONP-BBN recorded by Zetasizer device

By analyzing the size distribution and zeta potential, the Zetasizer device was used to confirm the cell’s interaction with nanoparticles that were impacted by its charge. The particle size distribution of IONP-BBN was displayed in Fig. 4a. It has demonstrated an average dynamic distribution of 246.3 nm particle diameters with 100% intensity. The zeta potential of IONP-BBN (Fig. 4b) exhibits a maximum value of − 28.7 mV. For a comparison the size distribution and zeta potential between IONP and BBN-IONP, according to a previous study, the average dynamic distribution of IONP is 1110 nm particle diameters with 100% intensity. While, the zeta potential of IONP exhibits a maximum value of -14.5 mV (Ibrahim Fouad et al. 2023). A significant variation in the particle size values was observed when TEM analysis and size distribution analysis was compared. This variation may have resulted from the agglomeration of nanopowders or from the different sample preparation techniques used for the two techniques (TEM and size distribution analysis) (Souza et al. 2016).

#### X-Ray photoelectron spectroscopy (XPS)

**Fig. 5:**
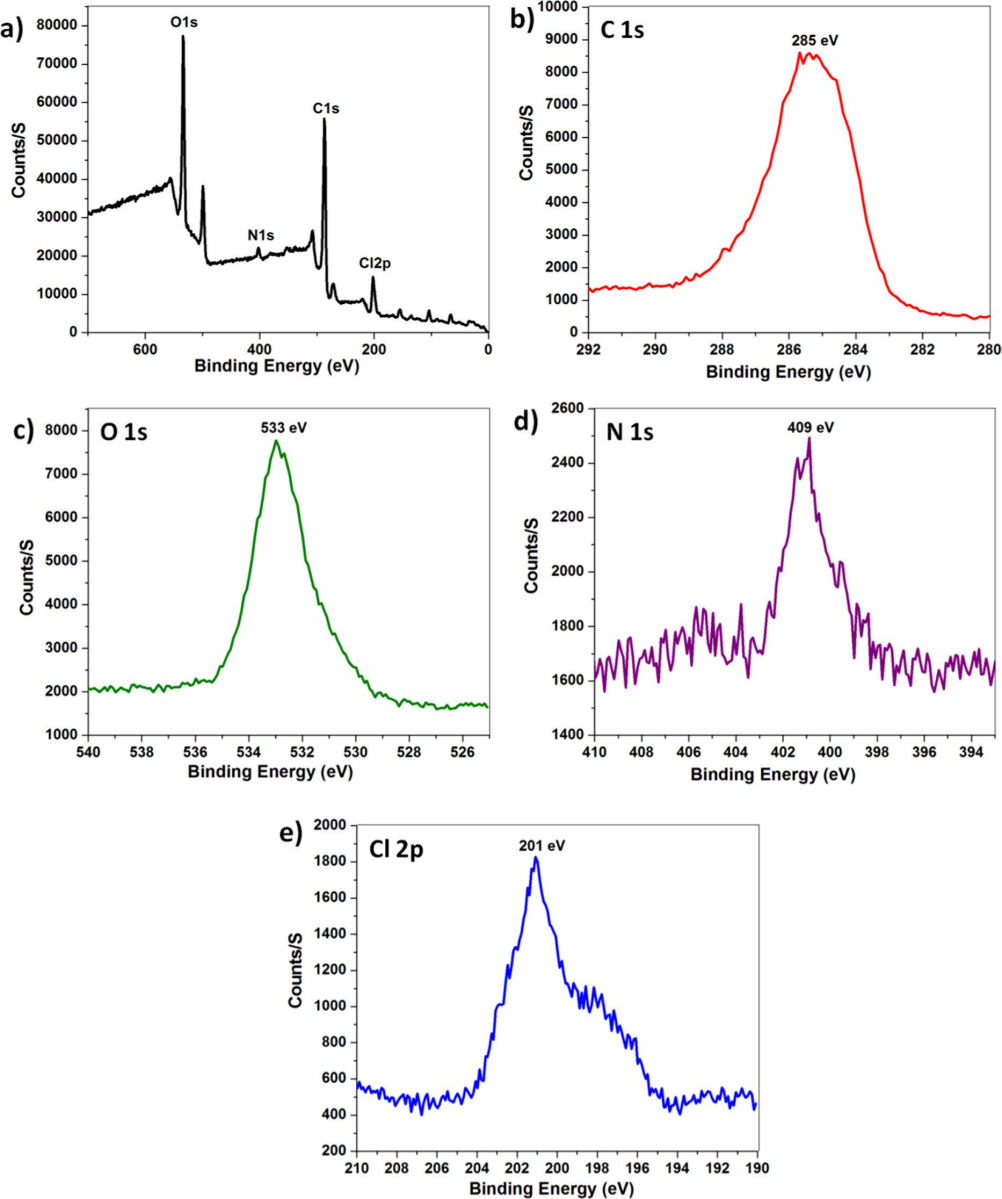
**a** XPS survey spectra of IONP-BBN, **b** High resolution C 1 s, **c** High resolution O 1 s, **d** High resolution N 1 s and **e** High resolution Cl 2p

With XPS analysis, the elements and functional groups present in a material, whether on its surface or within, might be quantitatively determined. The XPS survey spectra of IONP-BBN and the high resolution XPS spectra of C 1 s, O 1 s, N 1 s, and Cl 2p were displayed in Fig. 5. The XPS method verifies the components present in the medication berberine. The C 1 s spectrum corresponds to the binding energies of 285 eV. The binding energy of O, located at 533 eV in Fig. 5c for the O 1 s spectrum, confirms the presence of O. The N 1 s spectrum corresponds to the binding energies of 409 eV. Furthermore, Cl 2p’s spectra were detected around 201 eV (Liu et al. 2021).

#### HPLC analysis

**Fig. 6:**
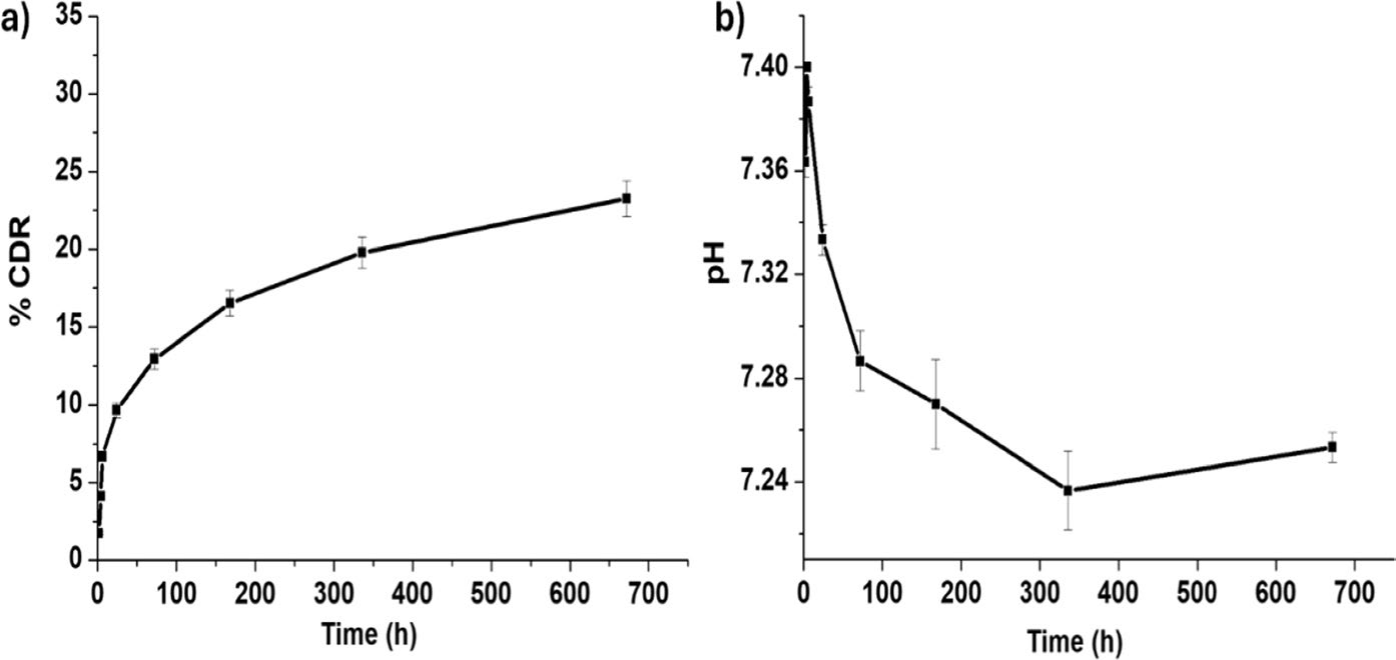
**a** Cumulative drug release (%) of berberine (BBN) from IONP-BBN and **b** pH of PBS at time intervals 2, 4, 6, 24, 72, 168, 336 and 672 h

The in vitro experiment measured and recorded the BBN concentrations using HPLC analysis. Figure 6a showed that the cumulative (%) drug release profile (% CDR) of BBN from IONP-BBN increased with time. The release profile showed that after 28 days, the drug was released approximately 25%. These results explored the significant role of IONPs, demonstrating their clear impact on the sustained release of BBN. Figure 6b shows the pH change that accompanied drug release. At the beginning, pH started at 7.4, and then underwent a sequential decrease up to approximately 7.2. The decrease in pH value was corresponding to BBN release, and this is attributed to the fact that BBN is a quaternary ammonium salt (Wang and Zidichouski 2018).

#### Biochemical results

##### Effect of free BBN and IONP-BBN on spatial working and reference memory in CPZ-neurotoxicated rats

##### a. Spontaneous alternation

There were no significant differences in the % of the alternation behavior among the different groups in experiment (1) of spontaneous alternation that measures spatial working memory, in Table 2.

**Table 2:** Comparing the Effect of free BBN and IONP-BBN on spatial working and reference memory in different experimental groups, using Y-maze test

Group	% of alterations	alterations	Novel arm	Start arm	Other	No. of entries
Negative	77.26 ± 3.69 ^b^	13.50 ± 0.65 ^c^	3 ± 0.26 ^b^	2.28 ± 0.36 ^a^	1.50 ± 0.18 ^a^	19.50 ± 0.65 ^b^
% of change to CPZ group	30.57	80	200	40.43	5.56	32.20
CPZ	59.19 ± 2.79 ^a^	7.50 ± 0.65 ^a^	1 ± .00 ^a^	1.96 ± 0.26 ^a^	1.58 ± 0.33 ^a^	14.75 ± 1.25 ^a^
% of change to control group	− 23.41	− 44.44	− 66.67	− 28.79	− 5.26	− 24.36
CPZ + BEB	73.23 ± 2.21 ^b^	11.50 ± 0.29 ^b^	3.17 ± 0.40 ^b^	2.83 ± 0.40 ^a^	2.17 ± 0.31 ^a^	17.75 ± 0.63 ^ab^
% of change to CPZ group	23.71	53.33	216.67	44.68	36.84	20.34
CPZ + IONP-BEB	74.76 ± 5.03 ^b^	11.25 ± 0.63 ^b^	3.50 ± 0.43 ^b^	2.33 ± 0.33 ^a^	1.67 ± 0.33 ^a^	17.25 ± 1.25 ^ab^
% of change to CPZ group	26.29	50	250	19.15	5.26	16.95

Differences between groups were analyzed using one-way ANOVA followed by Duncan’s post-hoc test. Data are expressed as mean ± standard error of the mean (SEM), (*n* = 8). Groups with similar letters are not significantly different; while those with different letters are significantly different at *p* ≤ 0.05. Mean with different superscripts (a, b) is significant at *p* ≤ 0.05

##### b. Spatial reference memory

In experiment (2) of spatial reference memory, CPZ-fed rats exhibited worse spatial memory, than the negative control group; they were much less able to tell the difference between new and old arms. On the other side, treated CPZ-fed rats with either free BBN or IONP-BBN demonstrated a good spatial memory, as compared to the CPZ-induced group, where rats could significantly differentiate between arms and achieve the maximum entries in the novel one (Table 2).

##### Effect of free BBN and IONP-BBN on LPO, TAC, and GSH in CPZ-neurotoxicated rats

**Fig. 7:**
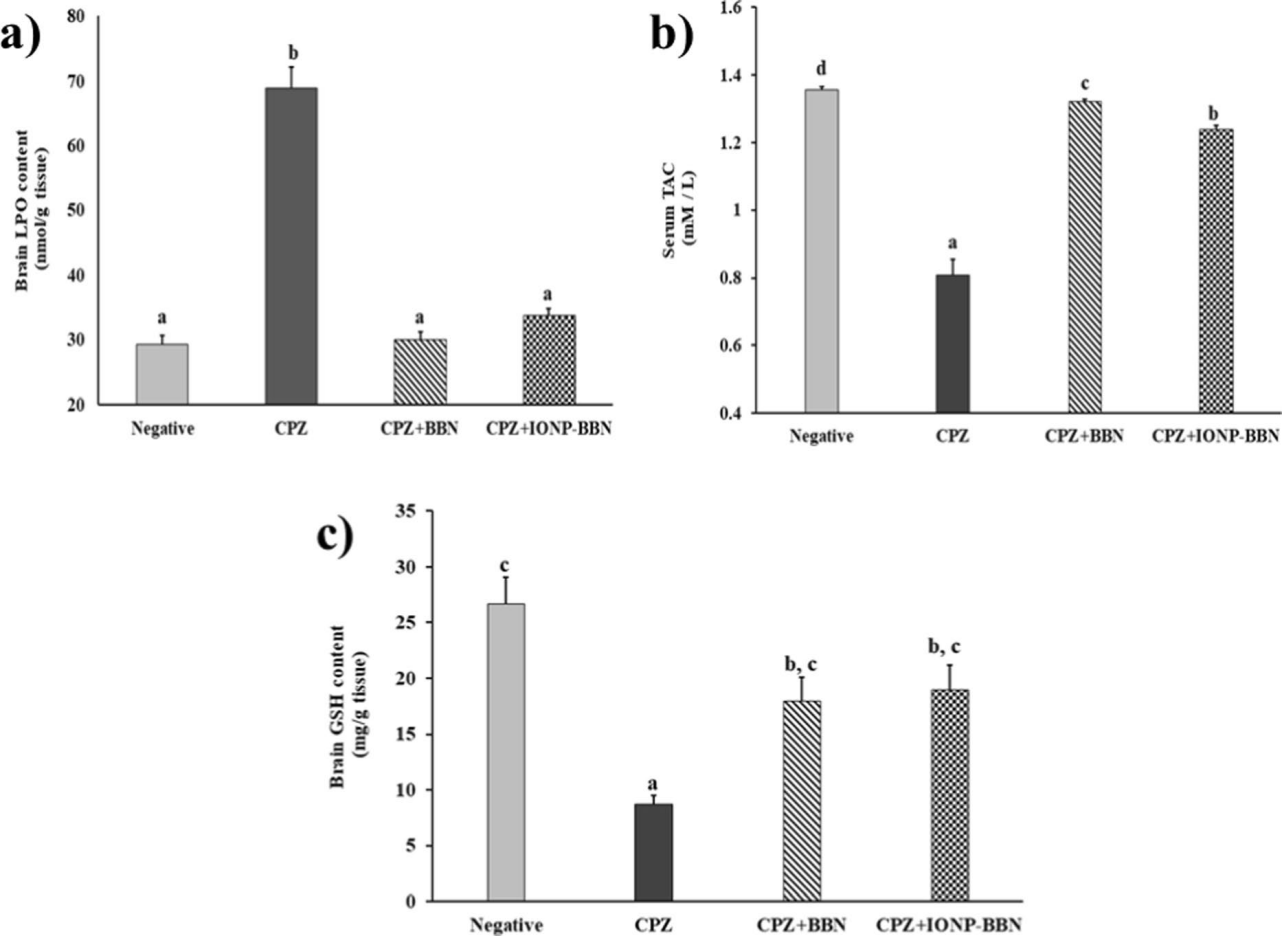
Comparing the anti-oxidative activities of Berberine (BBN) or BBN-loaded iron oxide nanoparticles (IONP-BBN) on cuprizone (CPZ)-induced oxidative stress in rats. Differences between groups were analyzed using one-way ANOVA followed by Duncan’s post-hoc test. Data are expressed as mean ± standard error of the mean (SEM), (*n* = 8). The mean with different superscripts **a–d** is significant at *p* ≤ 0.05. TAC: total antioxidant capacity, LPO: Lipid peroxide, and GSH: Glutathione reduced

As compared to negative control rats, CPZ-rats exhibited a significant increment in brain LPO by 135.2%, whereas serum TAC and brain GSH were significantly declined by 40.47% and 67.49%, respectively. On the other side, administration of either free BBN or IONP-BBN to CPZ-fed rats showed a significant decline in brain LPO by 56.36 and 50.9% respectively, and a significant increase in TAC and GSH by (63.86 and 107.61%) for BBN and (53.78 and 119.15%) for IONP-BBN, as compared to CPZ-neurotoxicated rats. These data revealed that both BBN and IONP-BBN exerted anti-oxidative activities against CPZ-provoked oxidative stress and supported the safe toxicological nature of IONPs (Fig. 7).

##### Effect of free BBN and IONP-BBN on IFN-γ levels in CPZ-neurotoxicated rats

**Fig. 8:**
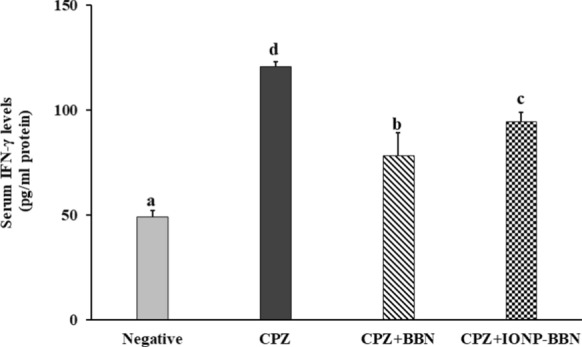
Comparing the immunomodulating activities of Berberine (BBN) or BBN-loaded iron oxide nanoparticles (IONP-BBN) on cuprizone (CPZ)-induced oxidative stress in rats. Differences between groups were analyzed using one-way ANOVA followed by Duncan’s post-hoc test. Data are expressed as mean ± standard error of the mean (SEM), (*n* = 8). Mean with different superscripts **a–d** is significant at *p* ≤ 0.05. IFN-γ: Interferon gamma

Dietary CPZ intake caused a significant elevation in serum IFN-γ level (145.16%), as compared to negative control rats. Treatment of CPZ-fed rats with BBN and IONP-BBN reversed the elevation in IFN-γ by (35.07%) for BBN, and (21.49%) for IONP-BBN, as compared to CPZ-rats (Fig. 8). These data revealed that both BBN and IONP-BBN exerted anti-inflammatory and immunomodulating activities against CPZ-provoked neuroinflammation.

##### Effect of free BBN and IONP-BBN on genetic expression contents of MBP, MMP-9, TNF-α, and S100β in CPZ-neurotoxicated rats

**Fig. 9:**
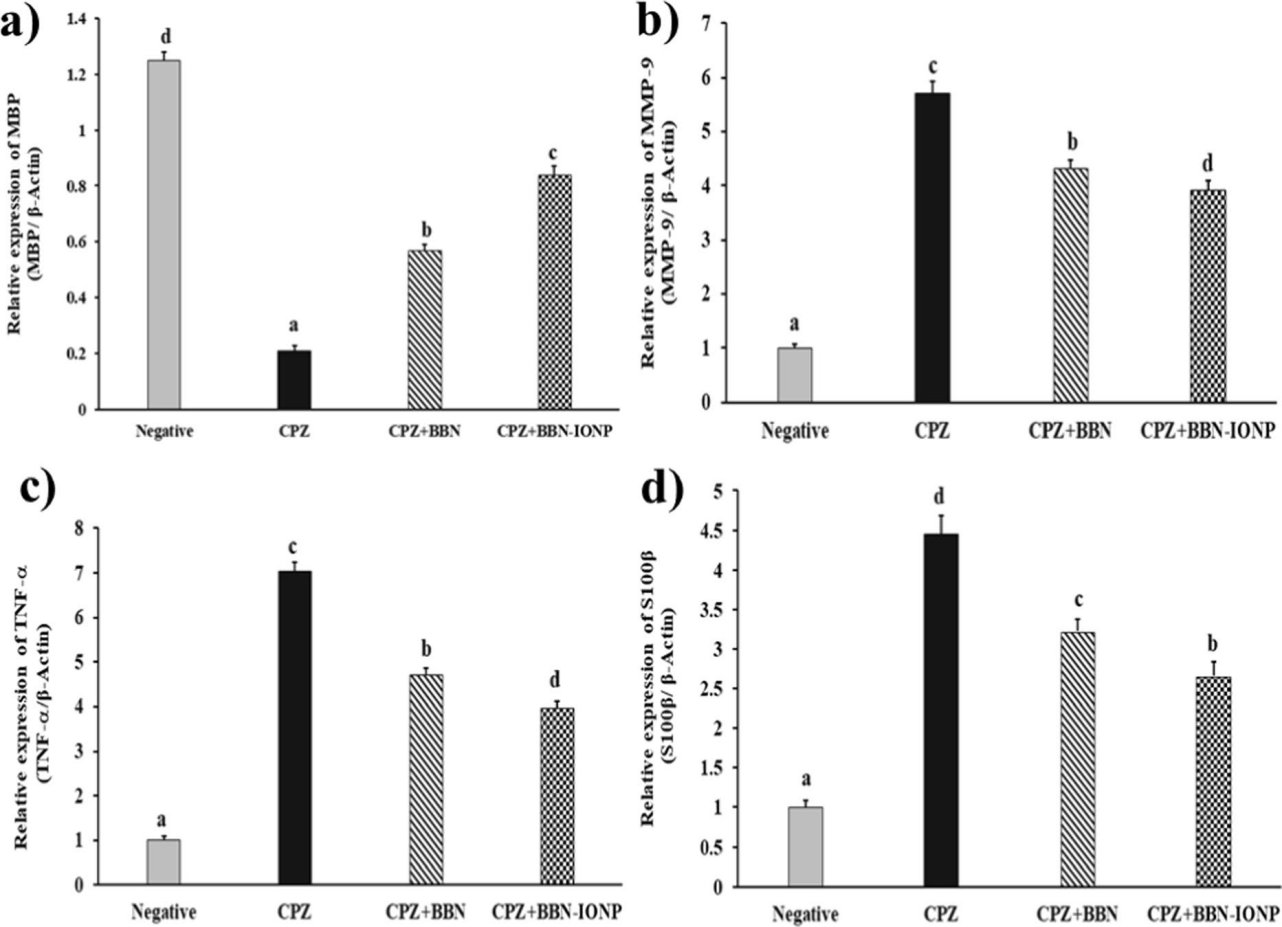
Comparing the pro-myelinating and anti-inflammatory activities of Berberine (BBN) or BBN-loaded iron oxide nanoparticles (IONP-BBN) on cuprizone (CPZ)-induced oxidative stress in rats. Differences between groups were analyzed using one-way ANOVA followed by Duncan’s post-hoc test. Data are expressed as mean ± standard error of the mean (SEM), (*n* = 4). Mean with different superscripts **a–d** is significant at *p* ≤ 0.05. MBP: myelin basic protein, MMP-9: Matrix metallopeptidase-9, TNF-α: Tumor necrosis factor alpha, and S100β: S100β-calcium binding protein

Genetic expression (cortical) contents of brain MBP, MMP-9, TNF-α, and S100β were estimated by qRT-PCR. As compared to control-diet fed rats, CPZ intake enhanced a significant downregulation of MBP expression (83%), along with significant upregulation of the expression of MMP-9, TNF-α, and S100β (470, 603, and 345%, respectively). While treatment with either free BBN or IONP-BBN significantly upregulated mRNA expression of MBP (175.11 and 305.58%, respectively), and downregulated the expression of MMP-9 (24.39 and 31.38%, respectively), TNF-α (32.79 and 43.62%, respectively), and S100β (27.47 and 40.26%, respectively), as compared to CPZ-induced brains. These data demonstrated that CPZ resulted in the down-regulation of mRNA levels of MBP expression (a marker of oligodendrocyte maturity), along with the up-regulation of mRNA levels of MMP-9, TNF-α, and S100β (markers of inflammation) that are related to MS; treatment of CPZ-rats with IONP-BBN was more effective in downregulating MS-related genes than free BBN (Fig. 9).

##### Histopathological results

### Effect of free BBN and IONP-BBN on the histopathological changes in cerebral cortices and hippocampi in CPZ-neurotoxicated rats

#### A) Microscopic examination of cerebral cortices from different experimental groups

**Fig. 10:**
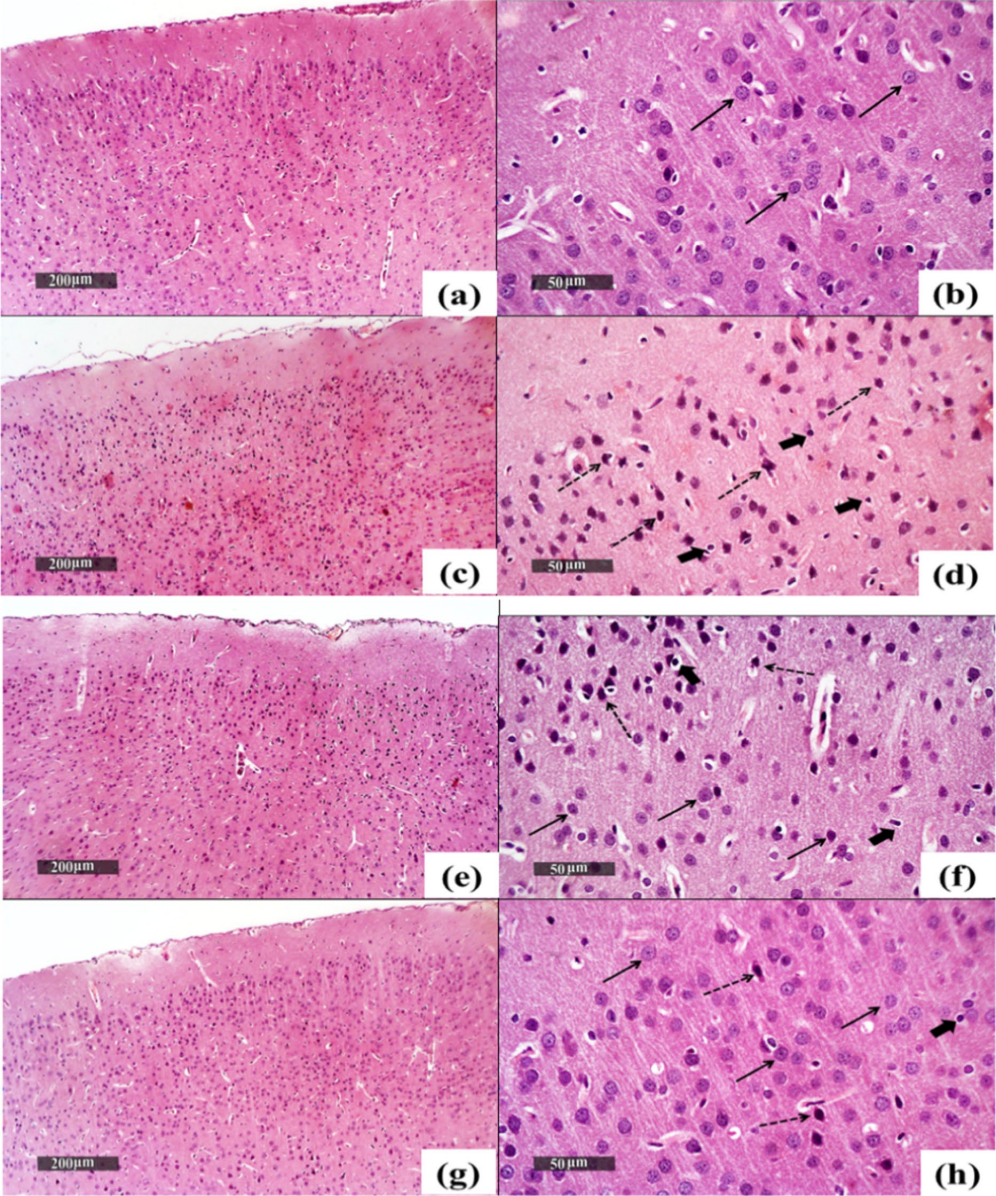
The structural alterations in the cerebral cortices of different experimental groups. Normal Control brains **a**, **b** exhibited intact and well-organized neural cells in different cortical layers (black arrow). CPZ-exposed cortices **c**, **d** exhibited shrunken pyknotic neurons in the outer layers (dashed arrow) and few scattered intact cells (black arrow) with moderate-higher inflammatory infiltrates (thick arrow). CPZ + BBN treated cortices **e**, **f** exhibited degenerated pyknotic neurons (dashed arrow) alternated with higher records of apparent intact neurons (black arrow), and minimal reactive glial cells infiltrates (thick arrow). CPZ + BBN-IONP treated cortices **g**, **h** exhibited few scattered records of degenerated cells (dashed arrow) and several apparent intact cells (black arrow), with minimal reactive inflammatory infiltrates (thick arrow). CPZ: cuprizone, BBN: Berberine, IONP-BBN: BBN-loaded iron oxide nanoparticles

Figure 10 demonstrated the effect of the treatment of CPZ-rats with BBN or BBN-IONP on the cerebral cortex. Normal Control brains (a, b) demonstrated intact histological structures of cortical layers with apparent well-organized neural cells in different layers (black arrow). An intact intercellular matrix was noticed with minimal reactive inflammatory infiltrates. CPZ-exposed cortices (c, d) displayed severe neurodegeneration along with the presence of many shrunken pyknotic neurons in the outer layers (dashed arrow) alternated with few scattered intact cells (black arrow), with moderate-higher inflammatory infiltrates (thick arrow). CPZ + BBN treated cortices (e, f) demonstrated focal areas of degenerated, pyknotic neurons (dashed arrow) alternated with higher records of apparent intact neurons (black arrow) and minimal reactive glial cells infiltrates (thick arrow). CPZ + BBN-IONP treated cortices (g, h) showed more organized morphological features of cerebral cortical layers with few scattered records of degenerated cells (dashed arrow) and many figures of apparent intact cells (black arrow), with minimal reactive inflammatory infiltrates (thick arrow).

### B) Microscopic examination of Hippocampal CA1 regions from different experimental groups:

**Fig. 11:**
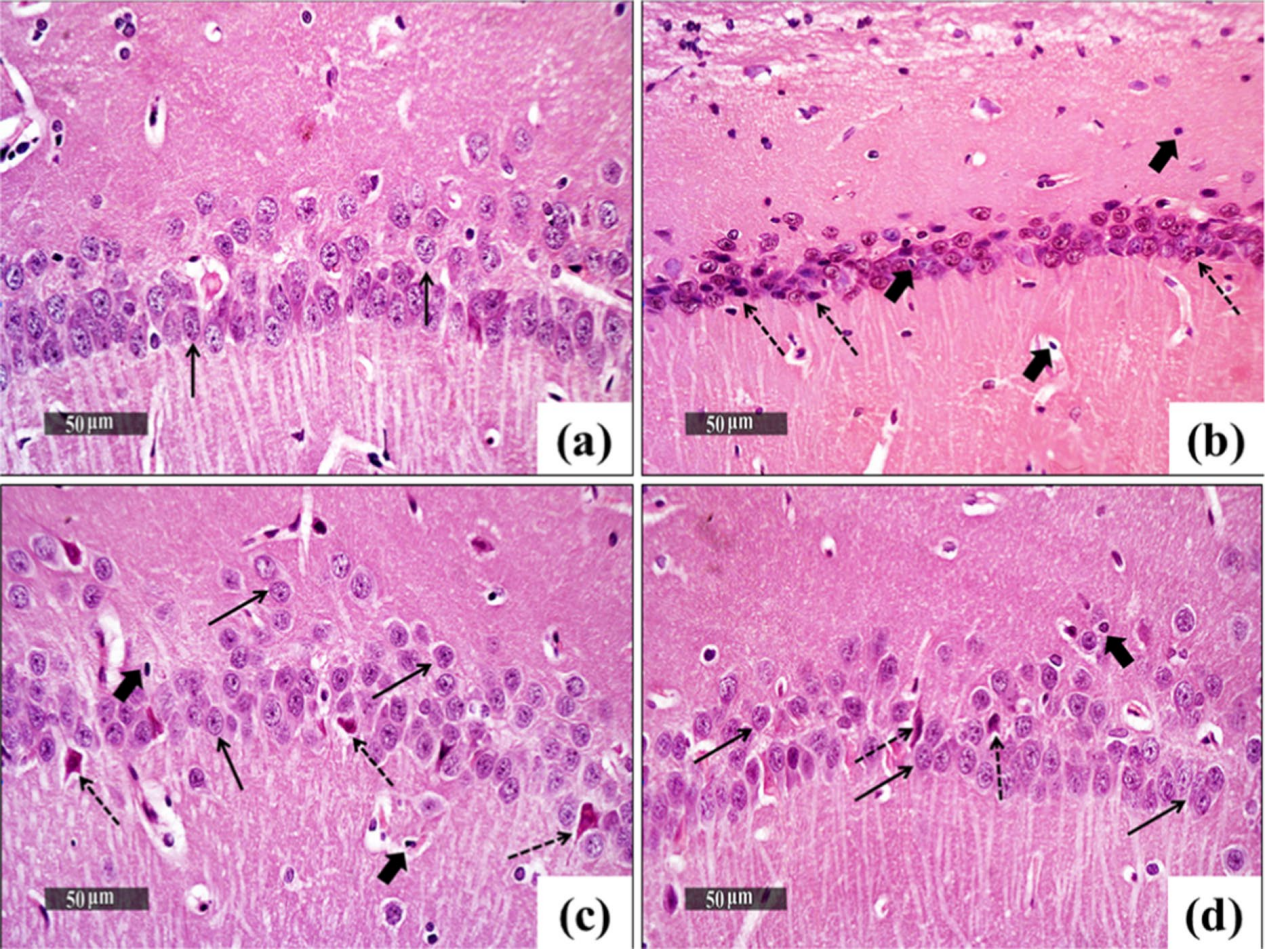
The structural alterations in the hippocampal CA1 regions of different experimental groups. **a** Normal Control hippocampi revealed normal and intact hippocampal layers with well-organized pyramidal neurons (black arrow). An intact intercellular matrix was noticed with minimal inflammatory infiltrates. **b** CPZ-exposed hippocampi showed moderate neuronal loss and abundant records of pyknotic neurons without distinct subcellular details (dashed arrow) alternated with a few scattered intact cells (black arrow) accompanied by moderate-higher inflammatory infiltrates (thick arrow). The hippocampi of the treated groups of **c** CPZ + BBN and **d** CPZ + BBN-IONP showed few scattered records of necrotic neurons (dashed arrow) alternated with higher records of intact neurons (black arrow) and milder inflammatory infiltrates (thick arrow). CPZ: cuprizone, BBN: Berberine, IONP-BBN**:** BBN-loaded iron oxide nanoparticles

Figure 11 demonstrated the effect of the treatment of CPZ-rats with BBN or BBN-IONP on the hippocampal CA1 regions. Normal Control hippocampi (a) revealed normal and intact hippocampal layers with well-organized pyramidal neurons (black arrow). An intact intercellular matrix was noticed with minimal inflammatory infiltrates. CPZ-exposed hippocampi (b) showed moderate neuronal loss and many pyknotic neurons without distinct subcellular details (dashed arrow) alternated with a few scattered intact cells (black arrow) and accompanied by inflammatory infiltrates (thick arrow). CPZ + BBN treated hippocampi (c) showed few scattered records of necrotic neurons (dashed arrow) alternated with higher records of intact neurons (black arrow) and milder inflammatory infiltrates (thick arrow). CPZ + BBN-IONP treated hippocampi (d) showed almost the same records as CPZ + BBN treated hippocampi.

### C) Effect of free BBN and IONP-BBN on CPZ-mediated glial cell activation: Glial Fibrillary Acidic Protein (GFAP) immunoexpression in cerebral cortical region and hippocampal CA1 region

**Fig. 12:**
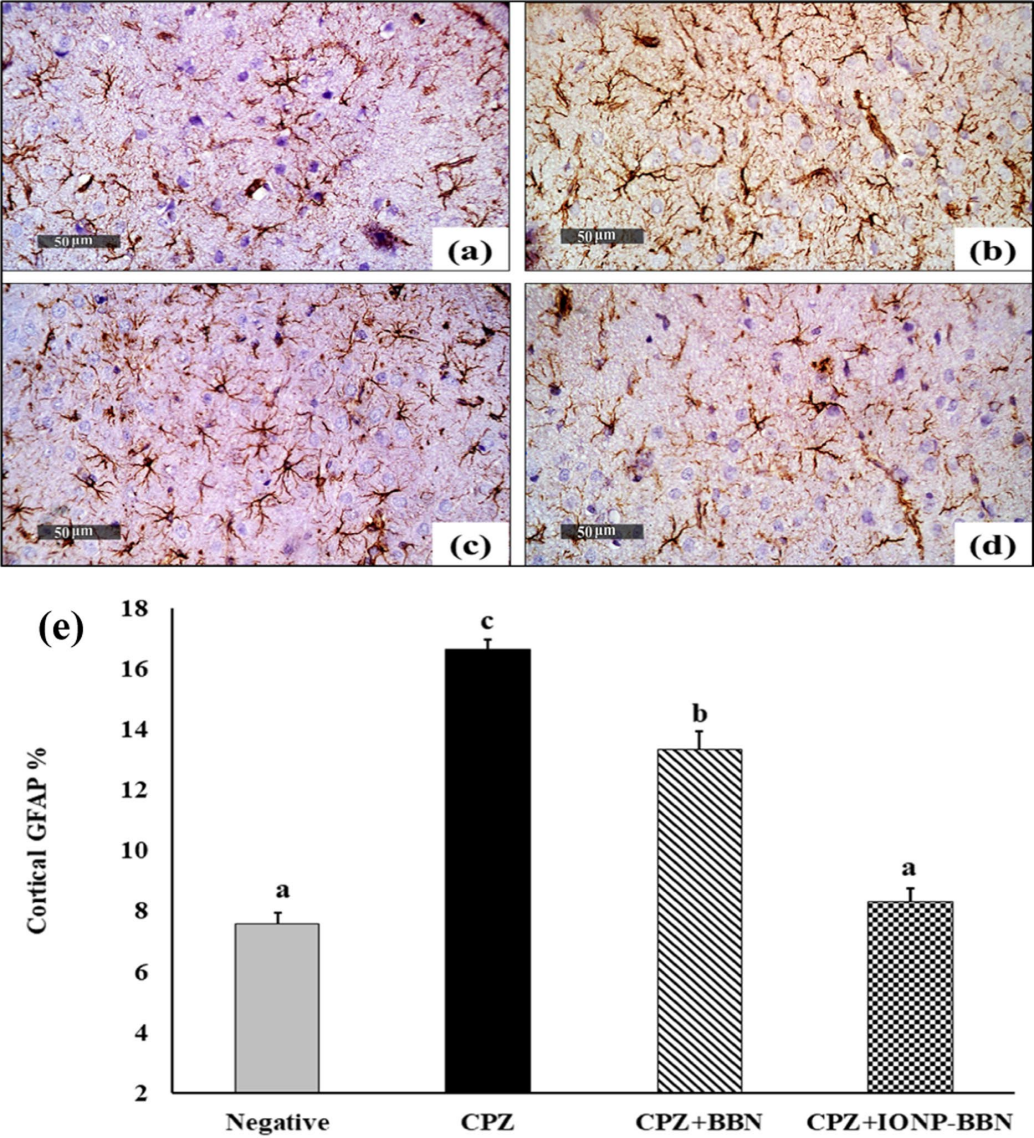
Comparing the re-myelinating and anti-inflammatory activities of Berberine (BBN) or BBN-loaded iron oxide nanoparticles (IONP-BBN) on cuprizone (CPZ) provoked immunoexpression of Glial fibrillary acidic protein (GFAP) in the cerebral cortexes. **a** Normal control cortex exhibited normal small-sized astrocytes with lightly-stained GFAP positive short processes. **b** CPZ-induced cortex exhibited strong immunoreactivity of astrocytes with deeply-stained GFAP positive brown processes. **c** CPZ + BBN-IONP cortex showing moderate GFAP positive immunoexpression. **d** CPZ + BBN-IONP cortex exhibited weak GFAP positive immunoexpression. **e** Percentage of immunostaining area of GFAP in the cerebral cortical regions of CPZ-induced brains. Differences between groups were analyzed using one-way ANOVA followed by Duncan’s post-hoc test. Data are expressed as mean ± standard error of the mean (SEM); (n = 6). Mean with different superscripts **a–c** is significant at *p* ≤ 0.05. *Preference for color: online*

**Fig.13:**
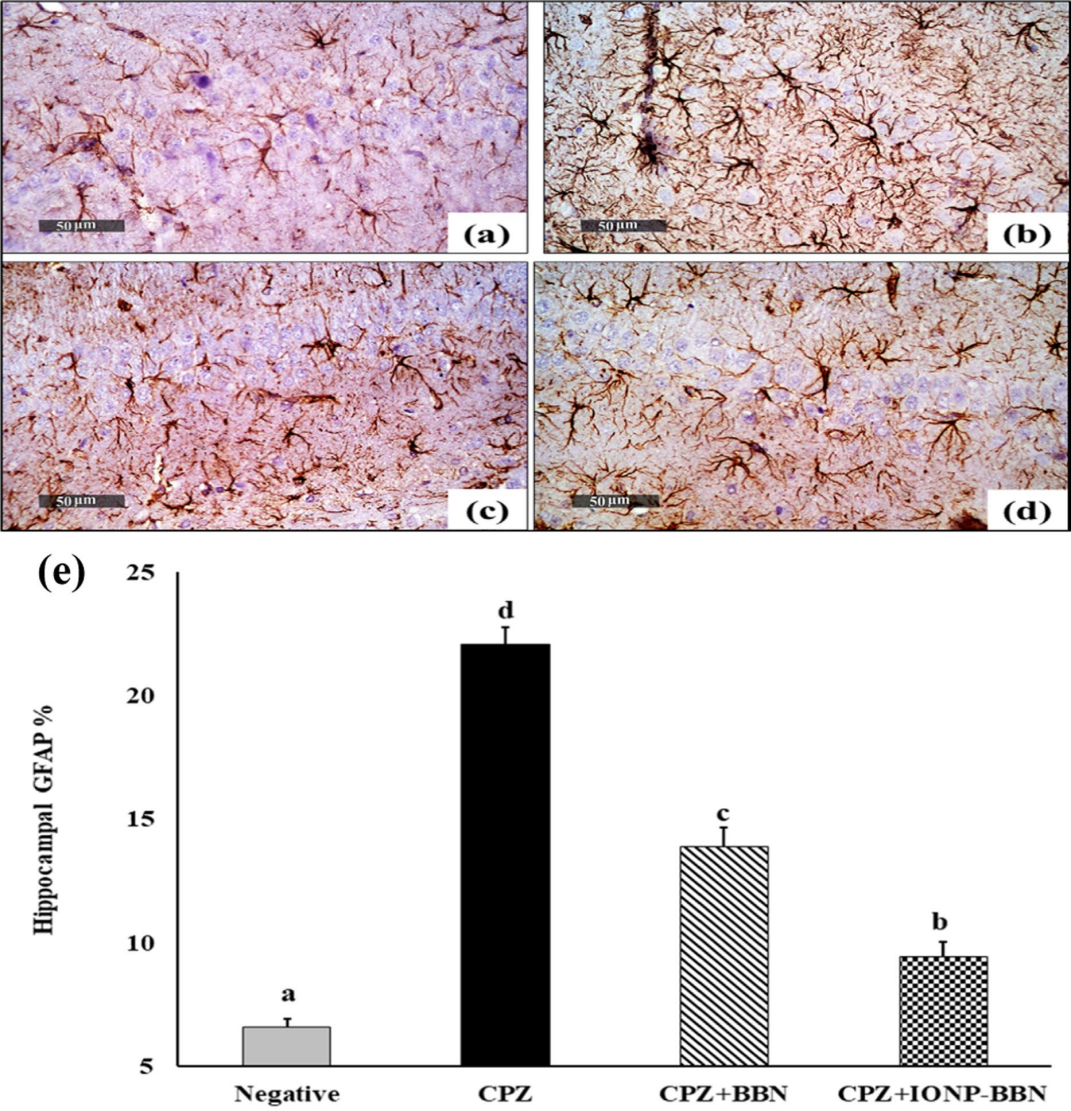
Comparing the re-myelinating and anti-inflammatory activities of Berberine (BBN) or BBN-loaded iron oxide nanoparticles (IONP-BBN) on cuprizone (CPZ) provoked immunoexpression of Glial fibrillary acidic protein (GFAP) in the hippocampal CA1 region. **a** Normal control hippocampus CA1 region exhibited normal small-sized astrocytes with lightly-stained GFAP positive short processes. **b** CPZ-induced hippocampus CA1 region exhibited strong immunoreactivity of astrocytes with deeply-stained GFAP positive brown processes. **c** CPZ + BBN-IONP hippocampus CA1 region showing moderate GFAP positive immunoexpression. **d** CPZ + BBN-IONP hippocampus CA1 region exhibited weak GFAP positive immunoexpression. e Percentage of immunostaining area of GFAP in the hippocampal CA1 region of CPZ-induced brains. Differences between groups were analyzed using one-way ANOVA followed by Duncan’s post-hoc test. Data are expressed as mean ± standard error of the mean (SEM); (n = 6). Mean with different superscripts (a-d) is significant at *p* ≤ 0.05. *Preference for color: online*

Figure 12 and 13 illustrated the comparison between the treatments of CPZ-intoxicated brain regions (cerebral cortex and hippocampus CA1) with either free BBN or IONP-BBN on the immunoexpression of GFAP. Microscopic investigation of negative control brains revealed normal small-sized astrocytes with lightly-stained GFAP + short processes (Fig. 12a, 13a). On contrary, strong immunoexpression of hypertrophied astrocytes with intensively brown-stained GFAP + processes was demonstrated in CPZ-neurotoxicated cerebral cortical region and hippocampal CA1 regions (Fig. 12b, 13b). On the other side, both the cerebral cortical region and hippocampal CA1 regions of CPZ + BBN recorded moderate immune-reactive astrocytes with lightly stained processes (Fig. 12c, 13c), while CPZ + BBN-IONP demonstrated weak immune-reactive astrocytes (Fig. 12d, 13d).

Figure 12e depicts the percentage area of GFAP immunoexpression in the cerebral cortices of different experimental groups. The area % of GFAP immunoexpression was significantly increased in CPZ-neurotoxicated brains (119.08%), as compared to control brains. Treatment of CPZ-fed rats with BBN and IONPs-BBN resulted in a significant decrease by 19.64 and 49.85% of GFAP area respectively, as compared to CPZ-brains.

Figure 13e depicts the percentage area of GFAP immunoexpression in the CA1 hippocampal region of different experimental groups. The area % of GFAP immunoexpression was significantly increased in CPZ-neurotoxicated hippocampus (236%), as compared to control brains. Treatment of CPZ-fed rats with BBN and IONPs-BBN resulted in a significant decrease by 37 and 57.24% of GFAP area respectively, as compared to CPZ-brains.

## Discussion

Magnetic IONPs, in either the magnetite (Fe_3_O_4_) or the maghemite (Fe_2_O_3_) forms, exhibited low toxicity and high biocompatibility (Zhao et al. 2020). In addition, due to their biodegradability, IONPs do not stimulate oxidative stress, even at high doses, and are effectively cleared from the body (Zhao et al. 2020; Attia et al. 2022). Moreover, IONPs exhibit a flexible surface chemistry enabling the loading of different bioactive molecules onto the surface, forming a drug-conjugated IONPs targeted delivery system (Tran et al. 2012). Herein, we utilized BBN-IONP to deliver BBN to the CPZ-intoxicated brains to evaluate its neurotherapeutic activities.

Cuprizone (CPZ) is a well-known copper chelator that attacks matured oligodendrocytes (OLG) which are responsible for the myelination of neurons (Acs and Kalman 2012). CPZ-induced copper decrement negatively impact the mitochondrial function, and subsequently impairing the antioxidant system, leading to demyelination (Largani et al. 2019). Intoxication with CPZ provoked oxidative stress in the brain of CPZ-induced rats, manifested as significant elevation in MDA (LPO), as well as, significant decrement of serum TAC levels and brain GSH contents. Neural tissue is highly vulnerable to oxidative stress; due to its relatively high oxygen consumption, elevated content of polyunsaturated fat and low anti-oxidant enzymatic activities (Fouad 2020). Normally, endogenous anti-oxidant GSH is implicated in mitigating oxidative stress through detoxifying free radicals (Pocernich et al. 2012).

Our findings run in accordance with several studies (Elbaz et al. 2018; Omotoso et al. 2019; Ibrahim Fouad and Ahmed 2023). CPZ-induced oxidative stress in the brain exhibits a relatively selective detrimental effect on oligodendrocytes (Ludwin et al. 1978), and initiates a cascade of pathogenic events including oligodendrocyte apoptosis, microglial and astroglial activation, and demyelination; resulting ultimately in MS-resembling pathology in animals (Acs and Kalman 2012; Praet et al. 2014; Omotoso et al. 2018). Histopathological findings of the hippocampal CA1 region and the cerebral cortex of CPZ-exposed brains support the biochemical results that CPZ exposure provokes oxidative stress in different regions of the brain. Our results run in agreement with Omotoso et al. (2018) who observed pyknotic cellular clusters in the CPZ-exposed hippocampi.

Behaviorally, Y-maze is used to estimate the “% alternation” to evaluate the working memory (Lassmann 2013). The decline in the memory recalling capacity revealed the neurotoxic potential of CPZ on the hippocampus, which is responsible for memory formation (Ogunlade et al. 2021). The decrease in synaptic connections directly correlates with neuroinflammation, leading to memory dysfunction (Hong et al. 2016). One of the features of the behavioral alterations in the CPZ-model of MS is hyperactive locomotion, as revealed by the total number of Y-maze arm entrances (Chang et al. 2017; Omotoso et al. 2018; Kopanitsa et al. 2021). The study by Kopanitsa et al. (2021) showed multiple alterations of spontaneous behavior and learning parameters in CPZ-induced mice. Similarly, Omotoso et al. (2018) demonstrated that CPZ intake enhanced spontaneous alternation deficits in Y-maze test. The impaired performance noticed in the activities of the CPZ-treated rats may be ascribed to the presence of scars in the anterior prefrontal cortex, and supports the existing connection between CPZ intake and decreased performances; neurocognitive function, and exploratory behavior, dependent on the intact integrity of the myelin sheath.

On the other side, treatment of CPZ-rats with either BBN or BBN-IONP showed anti-oxidative potential through lowering lipid peroxidation and raising brain GSH content and serum TAC levels, and thus conferred protection of neuronal function and integrity from oxidative insults. Reactive oxygen species (ROS) generation is related to GSH production; excessive ROS generation suppresses the anti-oxidant activity and reduces GSH levels (Li et al. 2024); however BBN demonstrated anti-oxidative potential against CPZ-induced oxidative stress (Sun et al. 2017; Cheng et al. 2022), this potential could be attributed to the activation of Adenosine monophosphate (AMP)-activated protein kinase (AMPK) and sirtuin1 (SIRT1)/forkhead box O1 (FOXO1) pathways (Li 2024). In addition, our previous study demonstrated that BBN exerted neuroprotective activity against doxorubicin-induced neurotoxicity in rats (Ibrahim Fouad and Ahmed 2021b); and therefore providing an evidence on the neuroprotective potential of BBN on different brain regions, however, the potential of BBN-IONP was more potent than that of molecular “free” BBN; histopathologically, we could observe that the neuroprotective potential of BBN-IONP was more obvious in the cerebral cortex of CPZ-brains than in the hippocampus. Previous studies reported that BBN-loaded nano-complexes demonstrated higher ROS-scavenging activity that leads to significant anti-oxidant potential (Bakshi et al. 2023; Mehra et al. 2024).

It is worth to mention that IONPs’ toxicity threshold can change based on their size. For instance, a study by Wu et al. (2022) demonstrated that when administered to mice at a dosage of 100 mg/kg, ultra-small IONP (diameters ranging from 2.3 to 4.2 nm) can be extremely toxic and fata1, on the other hand, at the same dosage, bigger IONP (about 9.3 nm) did not exhibit any obvious toxicity. This size-dependent in vivo toxicity of ultra-small IONPs could be ascribed to their potential to induce oxidative stress and ferroptosis in different organs, reflecting their good penetration of cell membranes.

Furthermore, CPZ administration affected the transcription of the major myelin protein-coding gene “MBP”. MBPis the most abundant protein in the myelin sheath (Frid et al. 2015). Our results run in agreement with Yu et al. (2018), Ohgomori and Jinno (2019), and Safwat et al. (2023) who found that CPZ administration resulted in a significant down-regulation of MBP “the mature OLGmarker” in the brain, as compared to control brains; indicating the neurotoxic and demyelinating potentials of CPZ through disruption of oligodendrocytes. Suppression of MBP expression might disrupt myelin synthesis and stability, leading to demyelination (Frid et al. 2015). On the other hand, qRT-PCR results indicated that both BBN and BBN-IONP treatments significantly upregulated the MBPexpression, as compared to CPZ-induced group.

Concerning neuroinflammation, CPZ administration resulted in significant upregulation of inflammatory markers (MMP-9 and TNF-α) and significant elevated serum levels of IFN-γ. MMP-9 activates several inflammatory mediators, contributes to the disruption of BBB’s permeability, and mediates the release of leukocytes in the brain parenchyma (Zirngibl et al. 2022; Beckmann et al. 2023; Safwat et al. 2023). Oxidative stress stimulates the expression of MMP-9 (Bai et al. 2006) and activates transcription factors, such as nuclear transcription factor-kappa β (NF-kβ), which upregulates the expression of inflammatory genes involved in MS, such as TNF-α (Ibrahim Fouad et al. 2023; Nicola et al. 2024). In addition, the digestive potential of MMP-9 is capable of destroying MBP (Safwat et al. 2023). Furthermore, Ma et al. (2010) found that upregulated expression of the latent-form of MMP-9 in the brain of autoimmune encephalomyelitis (EAE)-induced demyelination in mice, and the changes in MMP-9 activity in the cerebrospinal fluid (CSF) were linked to the expression of the protein. This study demonstrated the neurotoxic potential of CPZ to enhance neuroinflammation such as elevated brain TNF-α expression, as evidenced by previous studies (Sui et al. 2019; Yin et al. 2020; Safwat et al. 2023; Nicola et al. 2024). Microglia develops different functional phenotypes of pro-inflammatory (M1) that are capable of generating pro-inflammatory mediators, such as TNF-α (Hashimoto et al. 2017). Inhibition of TNF-α significantly mitigates the induced neurotoxicity, while the overexpression of TNF-α aggravates neural damage (Yang et al. 2017). Furthermore, CPZ-neurotoxicated rats exhibited a significant elevation of the pro-inflammatory IFN-γ; which is a cytokine produced mainly by T-lymphocytes, this runs in accordance with our previous study that demonstrated upregulated IFN-γ in inflammatory disorders (Ibrahim Fouad 2023).

In contrast, both BBN and BBN-IONP exerted anti-inflammatory potential by down-regulating the expression of both MMP-9 and TNF-α, through mitogen-activated protein kinase (MAPK) and NF-kβ signaling pathways (Ma et al. 2010). BBN could reduce the permeability of the BBB and downregulates the expression of MMP-9 in EAE mice and could suppress the expression of both MMP-2 and MMP-9 in vitro (Ma et al. 2010).

Astrocytes, the most abundant cell type in the CNS, have different functions to maintain the neural homeostasis under physiological conditions (Volterra and Meldolesi 2005). Under neurotoxic conditions, the activated forms of astrocytes generate a state of hypertrophic or reactive astrogliosis (astrocytosis) and upregulate the production of GFAP, as a specific structural protein that is exclusively expressed in astrocytes (Sofroniew 2015), and release several inflammatory mediators, resulting in gliosis and subsequent neuroinflammation (Williams et al. 2007). GFAP is responsible for maintaining the motility, shape, and mechanical strength of astrocytes, and contributes to the BBB integrity and myelination; therefore, GFAP could be regarded as a marker of brain injury that is used to assess the therapeutic impact of the intervention (Ibrahim Fouad and Ahmed 2021b). Gliosis “activation of astrocytes and microglia” is regarded as a “static scar tissue”, is considered as an active component of the neuropathogenesis of CPZ-induced demyelination (Skripuletz et al. 2013).

Herein, the immunohistochemical GFAP expression was investigated to evaluate the degree of CPZ-induced neurotoxicity and astrogliosis. In the CPZ-neurotoxicated cerebral cortical and hippocampal CA1 regions, the immunostaining area % of GFAP significantly increased, as compared to control brains, indicating reactive astrogliosis. The abundance of glial filaments indicates that CPZ intoxication stimulates astrogliosis, and the high immunostaining area of GFAP is a good indicator of astrogliosis (Ibrahim Fouad and Ahmed 2021b). Our findings run in coincidence with previous studies of Ohgomori and Jinno (2019); Nack et al. (2019); An et al. (2020); Kim et al. (2020); Toomey et al. (2021), and Castillo-Rodriguez et al. (2022). Our results indicated that CPZ-induced neurotoxicity is associated with microglial and astrocytic activation in different brain regions, where astrocytes act as “enclosing demyelinated areas” leading to exacerbated neuroinflammation.

The elevated GFAP immunoreactivity in the hippocampal CA1 region (236%) is pointing to the stimulation of neuroinflammatory responses and postulates that astrocytes might be one of the main targets of CPZ neurotoxicity; glial cells in the hippocampus were more vulnerable than those in the cortex. CPZ upregulated the expression of GFAP in astrocytes, especially in areas with large amounts of white matter, like the *corpus callosum* (Ibrahim Fouad and Ahmed 2021b, 2023), astroglial activation and increased GFAP expression have increasingly been directly implicated the suppression of axonal regeneration in demyelinating CPZ-induced MS.

The S100-βeta (S100β) protein belongs to the “S100 calcium-binding proteins” involved in cellular growth and differentiation, S100β is expressed in oligodendroglial progenitor cells committed to differentiating into oligodendrocytes, the S100β expression in GFAP-positive cells correlates with their maturation into the astrocyte lineage (Deloulme et al. 2004). In addition, S100β might be involved in the neuronal plasticity (Lisachev et al. 2010); brain-derived neurotrophic factor (BDNF) upregulates the expression of S100β in astrocytes (Djalali et al. 2005). In CPZ-neurotoxicated brains, S100β was detected in most “GFAP-positive astrocytes” in the cerebral cortex and hippocampal CA3 region (Castillo-Rodriguez et al. 2022). In the present study, dietary CPZ intake enhanced the expression of S100β gene (344.85%), as compared to negative brains; CPZ-induced rats exhibited a promoted astrocytic reactivity, represented as strong immunoexpression of GFAP protein, along with stimulated production of inflammatory mediators such as TNF-α and increased serum levels of IFN-γ.

Furthermore, a recent study by Kang et al. (2024) investigated neurotoxic microgliosis in Alzheimer’s disease (AD). It revealed that “AD neuron-reactive astrocyte interaction” stimulates IFN-γ production and generation of free radicals, mainly H_2_O_2_, and subsequently activates pro-inflammatory microglia, which can exacerbate synaptic dysfunction, deposition of tau proteins, and finally neurodegeneration. This runs in agreement with our study that demonstrated the neurotoxic of CPZ to stimulate oxidative stress, promote the production of IFN-γ, and activate the microglia and the astrocytes, leading finally to neurotoxicity and demyelination.

CPZ induced neuroinflammatory status is represented by astroglial activation, stimulated GFAP immunoexpression, upregulation of TNF-α and S100β, and elevated serum IFN-γ levels. However, these inflammatory events were alleviated upon administration of either BBN or BBN-IONP to CPZ-rats; through mitigating CPZ-induced microglial and astrocytic activation and attenuating the immunoexpression of GFAP in the cerebral cortex by 19.64 and 37%, respectively, as compared to CPZ-group. While hippocampal GFAP expression was reduced by 49.85 and 57.24%, respectively for BBN and BBN-IONP. This data demonstrated the anti-inflammatory and the anti-astrocytic activities of BBN, either free or loaded, against CPZ-provoked neuroinflammation and reactive astrogliosis. It’s interesting that treatment with BBN-IONP was more effective at significantly downregulating GFAP immunoexpression; this could better mediate the axonal repair process and speed up the recovery of neuronal function and memory recalling.

Astrological activation, inflammation, and oxidative stress are major contributors to CPZ-induced demyelination. Therefore, BBN’s potential to suppress these neuropathogenic mechanisms may confer protection against oxidative stress and inflammation in CPZ-neurotoxicated rats. BBN also exhibited immunomodulating potential through reducing serum levels of IFN-γ and downregulating the genetic expression of TNF-α and MMP-9 in treated CPZ-induced rats. This study indicated that BBN, in its free and nano- forms, mediated upregulation of MBP expression, and exhibited anti-inflammatory potential through suppressing the expression of MMP-9, that is responsible for digestion of MBP.

This neuroprotective potential might be ascribed to the ability of BBN to regulate miR-181c-5p/HMGB1 axis or to inhibit the HMGB1/TLR4/NF-κB pathway to alleviate inflammation (Cao et al. 2020), and to promote the transformation of activated microglia from the pro-inflammatory M1 phenotype to the anti-inflammatory M2 phenotype post-stroke (Zhu et al. 2018; 2019). Moreover, BBN could suppress astrocytic inflammation in diabetes-associated cognitive decline (Chen et al. 2017).

In the current study, treatment of CPZ-rats with BBN-IONP showed remarkable (superior) improvement in most of the biochemical, molecular, immunohistochemical, and histopathological studies, and demonstrated better efficacy in restoring the altered functional and structural activity of the selected panel of parameters. The nano-nature of BBN-IONP facilitates its ability to cross BBB, much more effectively and to exert its neuroprotective activities once it reaches an effective concentration in the brain. The use of nano-carriers to deliver bioactive natural molecules, such as polyphenols, enhances their bioavailability, retention time, and intracellular penetration; as revealed by the controlled delivery of those molecules (Santos et al. 2019). Thus, BBN-IONP can be utilized for controlled drug-delivery; thus reducing the side effects and frequency of administration of therapeutic agents.

It has been reported that the brain concentration of BBN are lower than that in other tissues after oral administration (Tan et al. 2013). Animal experiments showed that oral administration of BBN is safe, while intravenous or intraperitoneal injections are toxic and could induce multiple “dose- and time-dependent” toxic effects; such as neurotoxicity and cardiotoxicity (Rad et al. 2017; Tian et al. 2023). This toxic potential of BBN might be related to its ability of directly cross-linking with DNA or suppressing certain signaling pathways (Rad et al. 2017; Singh and Sharma 2018). Orally administrated BBN, with 100 mg/kg, has an absolute bioavailability of 0.68% as measured in rat plasma samples (Chen et al. 2011). In rats, the concentration of BBN in tissues was higher than plasma 4 h after oral administration at a dose of 200 mg/kg (Tan et al. 2013). At 200 mg/kg, oral administration of BBN demonstrated obvious cardioprotective and neuroprotective activities in rats (Geng et al. 2016; Zhao et al. 2019). Therefore, investigations on the in vivo cumulative toxicity is needed; more research should aim at investigating the pharmacokinetic and metabolic profile of BBN’s to reveal its metabolism, biodistribution, and absorption and identifying its ideal dosage to avoid side-effects associated with its accumulation (Ai et al. 2021). Further studies on BBN’s mechanism of action, as well as, novel formulations are therefore required.

## Strengths and Limitations

Due to their remarkable physiochemical properties, this study demonstrated that IONPs could be used as a safe and effective drug delivery system with a safe toxicological profile. The current research exhibits notable strengths, such as the innovative application of IONPs to improve the bioavailability of BBN, thorough characterization techniques (*e.g.*, TEM, FTIR, HPLC), and a pertinent CPZ-induced model that accurately simulates the demyelinating disorder of MS. Nonetheless, it possesses limitations, including a very small sample size, a brief treatment period of 14 days that may not adequately reflect long-term effects, and the absence of human trials restricting therapeutic relevance. The conventional use of IONPs for drug delivery might have some limitations, such as iron overdose and poor drug-loading efficiency, in addition, the lack of biodistribution and uncontrollable drug release features might impact the delivery of therapeutic molecules (Ezealigo et al. 2021). In addition, knowing the fate of IONPs might be challenging regarding the potential nanotoxicity after introducing NPs into the biological system; therefore utilizing imaging “tunable” NPs and a direct drug delivery (DDT) approach are required.

More preclinical studies are required to investigate the sex-specific biological mechanisms in the pathogenesis of the demyelinating disorder or the response to therapeutic interventions, through placing sex-dependent susceptibility as a biological variable that might have a key role in developing MS. In addition, evaluating the brain concentration of BBN should be conducted to explore the relation between the bioavailability of BBN in the brain and its neuroprotective activities, and investigate whether other factors might be involved. Therefore, future experimental studies are required to reveal the neuroprotective molecular mechanisms of BBN and to develop several nanoformulations of BBN to achieve the appropriate therapeutic BBN concentration with lower doses and better kinetic profile, along with optimizing its clinical efficacy, improving its bioavailability, and minimizing potential side effects.

## Conclusion

In conclusion, CPZ was able to induce demyelination, neuroinflammation, astrocytosis, and neurotoxicity, affecting both the hippocampus and the cerebral cortex. Conversely, the treatment with either BBN or BBN-IONP demonstrated neuroprotective potential by enhancing the anti-oxidant, anti-inflammatory, anti-astrocytic, and remyelinating activities. This also improved cognitive function, thereby maintaining the normal neuronal integrity that underpins brain functions. Loading BBN within IONPs altered the neuroprotective activities of BBN; resulting in superior neuroprotective potential of BBN-IONP relative to free BBN. This study supports the idea that this nano-based delivery system might be a promising neurotherapeutic approach that could promote the neurotherapeutic activities of BBN by improving its oral bioavailability and presenting sustained pharmacological levels of this polyphenol in the brain. Moreover, this study evidenced the potential of BBN to alleviate CPZ-induced neurotoxic astrogliosis.

## Data Availability

The authors confirm that the data supporting the findings of this study are available within the article.
